# Supplementary material for: From sequence to enzyme mechanism using multi-label machine learning
Source: BMC Bioinformatics. 2014 May 19;15:150. doi: 10.1186/1471-2105-15-150 (PMC4229970; doi:10.1186/1471-2105-15-150)
Supplement: Additional file 2 — Java code of ml2db. Additional file ml2db_code.tar.gz contains the Java source code to run the multi-label machine learning experiments and save the results to database. The code’s Javadoc is included. [file 1471-2105-15-150-S2.zip › additional file 2/ml2db/ml2db/doc/mulan/data/package-summary.html]

mulan.data


JavaScript is disabled on your browser.


- Overview
- Package
- Class
- Use
- Tree
- Deprecated
- Index
- Help

- Prev Package
- Next Package

- Frames
- No Frames

- All Classes

# Package mulan.data

- Interface Summary

  | Interface | Description |
  | --- | --- |
  |  |  |
  | --- | --- |
  | LabelClustering | An interface for various label clustering algorithms. |
  | LabelNode | Represents a label attribute as a node in the labels hierarchy. |
  | LabelPairsDependenceIdentifier | An interface for various types of dependency identification between pairs of labels. |
  | LabelsMetaData | Represents meta data about label attributes and their structure. |
  | Stratification | Interface for multi-label stratification methods |
- Class Summary

  | Class | Description |
  | --- | --- |
  |  |  |
  | --- | --- |
  | ConditionalDependenceIdentifier | A class for identification of conditional dependence between each pair of labels. |
  | ConverterCLUS | Class that converts a dataset that is originally in the format of the Clus system to a format that is suitable for Mulan. |
  | ConverterLibSVM | Class that converts LibSVM multi-label data sets to Mulan compatible format |
  | DataUtils | Utility class for data related manipulation functions. |
  | GreedyLabelClustering | A class for clustering dependent label pairs into disjoint subsets. |
  | IterativeStratification | Class for stratifying data based on the iterative stratification method |
  | LabelNodeImpl | Implementation of `LabelNode`, representing a label attribute and its connection within a hierarchy of labels. |
  | LabelPowersetStratification | Class for stratifying data based on label combinations |
  | LabelsBuilder | The `LabelsBuilder` is responsible for creation of `LabelsMetaDataImpl` instance from specified XML file source. |
  | LabelSet | Class that handles labelsets |
  | LabelsMetaDataImpl | Implementation of `LabelsMetaData` info about labels and their structure. |
  | LabelsPair | Class for handling label pairs along with their 'dependence' scores. |
  | MultiLabelInstances | Implements multi-label instances data set. |
  | Statistics | Class for calculating statistics of a multi-label dataset. |
  | UnconditionalChiSquareIdentifier | A class for identification of unconditional dependence between each pair of labels using Chi Square Test For Independence. |
- Exception Summary

  | Exception | Description |
  | --- | --- |
  |  |  |
  | --- | --- |
  | DataLoadException | The exception is thrown to indicate an error while loading the data. |
  | InvalidDataFormatException | The exception is thrown when format of the data is not valid. |
  | LabelsBuilderException | Exception is raised by `LabelsBuilder` to indicate an error when creating `LabelsMetaDataImpl` instance form specified source. |

- Overview
- Package
- Class
- Use
- Tree
- Deprecated
- Index
- Help

- Prev Package
- Next Package

- Frames
- No Frames

- All Classes
